# Impact of a surfer rescue training program in Australia and New Zealand: a mixed methods evaluation

**DOI:** 10.1186/s12889-023-17057-w

**Published:** 2023-11-08

**Authors:** William Koon, Amy E. Peden, Robert W. Brander

**Affiliations:** 1 School of Biological Earth and Environmental Sciences, University of New South Wales, Kensington NSW 2052, Sydney, NSW Australia; 2 Beach Safety Research Group, University of New South Wales, Sydney, NSW Australia; 3 School of Population Health, University of New South Wales, Sydney, NSW Australia

**Keywords:** Drowning prevention, Program evaluation, Safety intervention, Surfers, Ocean safety, Risk reduction, Community development

## Abstract

**Background:**

Surfers play a critical role in coastal drowning prevention, conservatively estimated to make as many rescues as beach lifeguards. The Surfer Rescue 24/7 (SR24/7) program is a coastal safety intervention in Australia and New Zealand that teaches surfers safe rescue skills and promotes prevention activities. This multi-part, mixed-methods study aimed to evaluate the impact of the SR24/7 program.

**Methods:**

The study consisted of three parts employing quantitative and qualitative methods: a retrospective survey of course participants, in-depth interviews with course participants who had conducted rescues, and an analysis of self-reported skills confidence ratings before and after the program.

**Results:**

Triangulated results from the three study components indicated that after the course, participants exhibited high levels of satisfaction with their experience in the program and would encourage others to attend, were more observant and aware of safety concerns while surfing, had a better understanding of ocean conditions and hazards, learned new rescue techniques and skills, grasped important course concepts related to their own personal safety, and improved their confidence in responding to an emergency situation. Several participants had conducted rescues in real life and indicated that the course was effective in providing them with the baseline knowledge and skills to keep safe while helping others in the ocean. This study also provides new insight on the role of surfers in coastal safety, specifically that surfers are engaged in a range of prevention activities before rescue is required.

**Conclusions:**

Despite persistent challenges in combating coastal drowning rates, the SR24/7 program is an effective intervention that helps save lives. Importantly, this study provides evidence that the course successfully equips surfers with techniques to act responsibly and safely. Expanding coastal safety focus and resources towards surfers, an often-overlooked demographic in beach safety strategies, could substantially enhance community-level capacity to prevent and respond to ocean emergencies.

**Supplementary Information:**

The online version contains supplementary material available at 10.1186/s12889-023-17057-w.

## Background

Drowning is a global health threat accounting for approximately 236,000 fatalities globally each year, though this burden is likely to be higher when water transport and disaster-related drownings are included [1, 2]. In addition to deaths, non-fatal incidents contribute to the substantial economic, societal, and emotional burden of drowning [3–5]. Consequently, there has been considerable multisectoral efforts to prevent drowning from a variety of perspectives including public health, leisure/sport, emergency services, transport, and education, among others [6]. The global drowning challenge was recently highlighted in a United Nations General Assembly Resolution, which renewed calls for the development of drowning prevention programs, including those focused on rescue and resuscitation training [7]. While drowning events occur in all bodies of water, coastal environments present particularly complex risk conditions which makes ensuring safety in these locations difficult [8].

Rescue and resuscitation are integral components in mitigating the impact of drowning [9] and have been prominently featured in models such as the Drowning Chain of Survival and the Drowning Timeline [10, 11]. Especially in the coastal environment, rescue is a particularly challenging component of prevention due to the risk of injury or death for the rescuer [12]. Typically, discussions on coastal/beach rescue focus on professionally trained lifeguards and other emergency responders as well as trained volunteer surf lifesavers in countries like Australia and New Zealand [13].

Bystander rescue (i.e., not from a trained lifeguard/lifesaver) has been comparatively less emphasized, although attention on the issue has increased in recent years [14]. Untrained bystander rescues are unfortunately the cause of multiple deaths each year in Australia [12, 15, 16] and other countries [17, 18]. Experts recommend that bystanders should attempt to help a drowning person *without* entering the water, for example by throwing or reaching to the person with a pole, rope, or flotation device [14]. However, there is exception to this guidance if the rescuer has “good aquatic competence, good physical fitness, good experience in the relevant aquatic environment, and some flotation equipment” [14]. Generally, recreational surfers fit these criteria.

Surfing involves an individual riding a buoyant board down the face a wave towards shore and is a popular recreational activity in coastal communities around the world with historical, cultural, and economic significance [19]. While the role of surfing and surfers in coastal safety is less emphasized than lifeguards and lifesavers, previous research from Australia conservatively estimated that recreational surfers conduct as many rescues annually as trained beach lifeguards and lifesavers [20], largely due to surfers being in the water and/or on the beach when and where lifeguards and lifesavers are not. For this reason, the World Health Organization identified surfers as a target population for safe rescue and resuscitation training programmes [21]. Furthermore, several studies on surfer rescues have called for basic lifesaving and cardiopulmonary resuscitation (CPR) training to be promoted within the surfing community as a measure to reduce drowning [20, 22–26].

Some coastal safety and ocean lifeguard organisations have expressed legitimate concern that inexperienced surfers might get themselves into trouble attempting rescues, thereby risking further loss of life and complicating beach safety management. Historical tension between surfers and lifeguards/lifesavers notwithstanding [27], formal rescue training programs for surfers have operated in Australia since at least 2012 and have subsequently expanded to other countries. While evidence exists justifying the rationale and recommendation of such programs, mainly that surfers are already conducting rescues [20, 23, 25, 26], their methods and impact have yet to be described or evaluated. Understanding what surfers learn, how their attitudes and beliefs are influenced, and ultimately how they change their actions based on their participation in a program serves to establish best practice for this innovative and potentially impactful coastal drowning prevention effort.

This research aims to evaluate the effectiveness and implementation of the Surfers Rescue 24/7 (SR24/7) program through a multi-part mixed-methods study. This will inform SR24/7 program upgrades, which may increase training quality and indirectly contribute to the reduction of drowning rates in this study context. Specific objectives of the project were to: 1) evaluate the impact of the course on participant confidence, knowledge, attitudes/beliefs, and self-reported skills and behaviours; 2) further characterize the role of surfers as community/bystander responders to coastal emergencies in Australia and New Zealand; and 3) establish and disseminate best practices for enhancing existing rescue courses for surfers and developing new ones, ensuring their expanded application and efficacy throughout Australia, New Zealand, and globally. Given the role that surfers have been shown to contribute to ocean rescue, this evaluation has global implications for mitigating the occurrence and consequences of coastal drowning incidents.

## Methods

### About the surfers rescue 24/7 (SR24/7) program

The SR24/7 program is a board rescue and CPR course specifically tailored for recreational surfers. Developed in 2012 by Surfing New South Wales (SNSW), the leading authority responsible for promoting competitive and recreational surfing in the state of New South Wales (NSW), Australia, the program has since garnered support from the NSW Government Water Safety Fund, world champion surfers like Kelly Slater, and was recognized as the Community Water Safety Education Program of the Year in 2019 [28]. SR24/7 provides essential training on how to safely assist individuals in the water using rescue techniques designed and endorsed by Australia's primary ocean rescue organizations, including Surf Life Saving Australia (SLSA) and the Australian Lifeguard Service (ALS).

Typically lasting about three hours, the free or low cost SR24/7 course consists of both theoretical and practical components. The theoretical portion is delivered in a classroom setting or on the beach, while the practical segment allows participants to practice various rescue techniques in the water under the supervision of experienced instructors. Additional information on the course, including photos and videos, is available at https://www.surfersrescue247.com. The program has expanded beyond NSW and is now offered in the Australian states of Victoria, Queensland, and Western Australia, as well as in New Zealand. Similar programs teaching basic rescue techniques and CPR to surfers are available in the United States [29], Brazil [30], and various parts of Europe [31, 32].

This study focused on SR24/7 programs conducted in: i) New South Wales (NSW) by Surfing New South Wales (SNSW); ii) Victoria by Surfing Victoria (SVIC); and iii) New Zealand (NZ) by Surfing New Zealand (SNZ).

### Study design

**Fig. 1:**
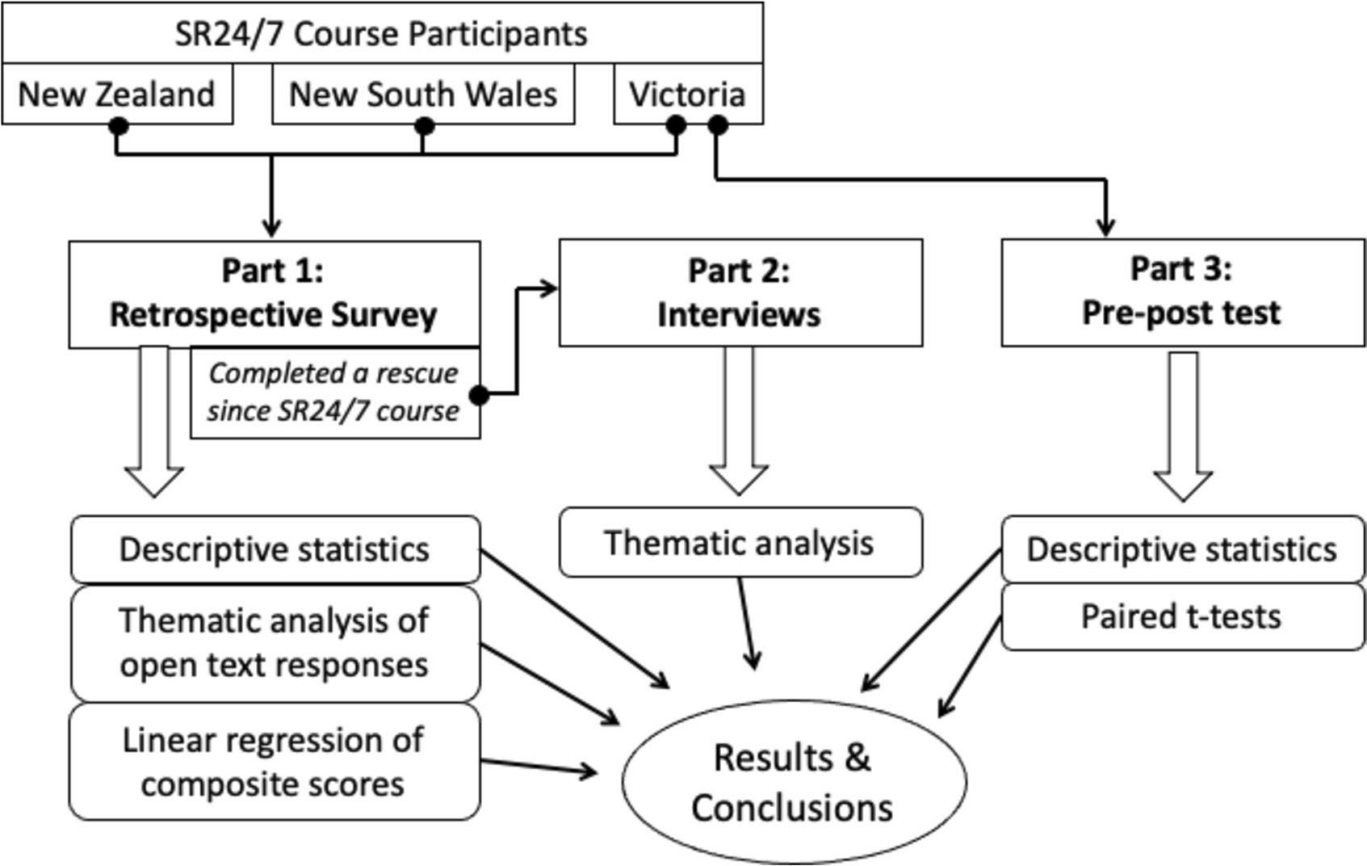
Multi-part mixed methods study design and analysis methods for SR24/7 program evaluation

This study is a multi-part mixed methods evaluation of the Surfers Rescue 24/7 (SR24/7) program that resembles a modified, type II hybrid mixed methods evaluation where quantitative and qualitative data are embedded and serve a complementary function as described by Palinkas et al. [33]. That is, the study aims examine both the effectiveness and implementation of the program equally and simultaneously, using both quantitative data, which provides information on outcomes and a breadth of understanding of the issue, and qualitative data, which explores implementation process and provides depth of understanding of the issue [33]. The study was broken into three parts: Part One involved a retrospective survey of participants that provided both quantitative and qualitative data on both program impact and implementation; Part Two included in-depth interviews, which produced rich qualitative data on participant’s experience in the course and as surfers who had conducted rescues in real life; and Part Three represented a quantitative pre-post analysis on participant’s confidence to perform a rescue and, separately, CPR (Fig. 1). Synergistically, this approach provides a better understanding of the issue and domain of inquiry than any single part could have alone [33].

### Part one: retrospective survey

Part One involved a retrospective, cross-sectional survey of past SR24/7 program participants from NSW, Victoria, and NZ, designed to assess attitudes and beliefs about the program and surfers' role in beach safety. The survey included process and personal satisfaction questions for quality improvement purposes and assessed if, and how, participants have used skills they learned in the program.

#### Participants and recruitment

Participants were recruited with the help of SNSW, SVIC, and SNZ, who supplied SR24/7 course records and/or contacted previous participants directly. Inclusion criteria for the retrospective survey were that, at the time of invite, the person was 18 years of age or older, and had previously completed a SR 24/7 Course in NSW, Victoria, or NZ in the past two years. From initial course rosters, previous participants were contacted via email with information outlining the research and inviting them to participate in the retrospective survey. Two follow up emails were sent to those who had not completed the survey. As an incentive, those who completed the survey entered a draw to win various gift certificates.

#### Data collection and instruments

Survey questions were constructed in consultation with SNSW and based on previous literature related to surfer rescue [20, 23]. The survey contained multiple choice, Likert-type, and open response questions (36 possible questions total) across eight themes: Screening and respondent background (nine questions), course satisfaction (three questions), course learning (six questions), knowledge check of course material (three questions), use and implementation of course skills/knowledge (nine questions), surfer role and responsibilities in coastal safety (four questions), confidence to perform a rescue (one question), and future opportunities (one question). For Likert-type questions, all but one had response categories following a seven-point scale from “1 – Strongly Disagree” to “7 – Strongly Agree”; one course satisfaction question asked the respondent to rate the Surfers 24/7 Course from “1 – Terrible” to “7 – Excellent”. A draft survey was piloted by SNSW staff, who provided feedback that was incorporated into the final survey, which was estimated to take between 10 and 15 min to complete. Surveys were administered online via the Qualtrics Survey Platform. The retrospective survey questions and relevant thematic groupings are available in Additional File 1.

#### Data analysis

We reported frequencies and descriptive statistics for multiple choice and individual Likert-type questions and created composite scores to evaluate respondent attitudes relevant to course satisfaction, course learning, and the role of surfers in coastal safety [34]. Composite scores were derived by calculating the mean of Likert-type questions for each attitude domain where lower scores reflected negative and higher scores reflected positive attitudes. If a respondent did not answer a question used in a composite score, their mean composite score was calculated excluding the skipped question; the impact of missing data on composite scores is likely minimal as the max number of missing responses in a question was 6 (2.5% of all responses received), the median number of missing responses in a question was two (0.8%). Some Likert-type questions included a possible response for “Don’t Know / Can’t Remember”, but this option was not selected in any question. Additional File 1 identifies which Likert-type questions were included in each composite score.

We reported means and evaluated for differences in composite scores by surf experience, surf ability, and surf frequency. As each of these variables are ordinal, where each response represents a greater than or less than relationship to the other response options in the question, we assigned numbers to each response option and evaluated for trends using simple linear regression [34]. 'Surf experience’ (number of years the respondent had been surfing) was grouped and assigned the following numbers: 0 – “Less than 1 year”, 1 – “1 to 5 years”, 2—“6 to 10 years”, 3 – “11 to 20 years”, and 4 – “21 years or more”. ‘Surf ability’ represents self-reported surf skills adapted from Berg et al., 2021 and was grouped and assigned the following numbers: 0 – “Novice/beginner”, 1 – “Intermediate”, 2 – “Advanced”, and 3 – “Expert/professional”. Surf frequency at the time of course was grouped and assigned the following numbers: 0 – “Less often”, 1 – “1 to 2 times per week”, 2 – “3 to 4 times per week”, and 3—“5 + days per week”.

The survey also included open response questions intended to: i) help improve understanding of the motivation for surfers to sign up to the program; and ii) identify strong elements of the course from the perspective of the course participant. Open text answers were analysed using a modified theoretical thematic analysis process [35]. WK read all answers, coded the responses, and defined initial themes. AP and RB also read all responses and reviewed the initial themes proposed by WK. The author team collectively discussed and refined the themes for final presentation.

Survey analysis was conducted in r Studio and Tableau Desktop [Version 2022.1]; open response questions were thematically analysed using NVivo 12 [Computer Software].

### Part two: interviews

Part Two involved semi-structured, in-depth interviews with previous course participants who had conducted a rescue in the ocean while surfing *after* their participation in a SR24/7 course. This approach was both descriptive in nature to explore emerging themes and sub-themes and involved more analytical processes to contribute to the theoretical understanding of motivators, facilitators, and barriers to participation in the SR/247 course and application of learning/lessons from the course in real life situations and rescues [35–37]. There is a well-established precedent for using semi-structured interviews in planning and evaluating health services and programs [38, 39], and more specifically water safety and drowning prevention programs [40, 41]. Engaging in this qualitative process was motivated by the need to identify elements of the SR24/7 program that surfers felt were important to a successful course and help further characterise the role of surfers as bystander rescuers, including their confidence to stay safe, willingness to help, and actual skills to assist the person in need.

#### Participants and recruitment

Potential participants for the interviews were identified from the past participant survey. Survey respondents who indicated they had conducted a rescue after their SR24/7 course were asked if they would be interested in discussing their experience in the course and making a rescue with a member of the research team. Those who responded “yes” (*n* = 26) received an email outlining the purpose and basic logistics of the interview and were provided a link to sign up for an interview time. After the initial interview invitation email, up to two follow up emails were sent. To encourage participation in the interview and compensate the participant’s time, interview participants received various gift vouchers via email upon completion of the interview.

#### Data collection

WK conducted 14 in depth interviews in July 2022, August 2022, and February 2023. Discussions followed an interview guide developed in consultation with SNSW and based on best practice for semi-structured interviews designed for program evaluation [42, 43]. The interview guide included key questions related to two domains: participant’s experience in the SR24/7 course and, separately, their experience rescuing other people at the beach while surfing (Additional File 2).

All interviews occurred via online video conferencing software and lasted between 25 and 45 min. With the participant’s consent, the interviews were recorded, and a text transcript of the interview was automatically generated by the video conference software. The software generated transcript was reviewed and checked for mistakes by WK, cross-referenced with the audio recording where appropriate. Identifying information from the transcript (e.g., the participant’s name) was removed, and the transcripts were sent to individual participants for member checking to provide an opportunity for corrections or additions. WK took notes during and after each interview for triangulation purposes. One interview participant did not consent to the interview being recorded, in this case, WK’s interview notes were sent to the participant for member checking and formed the qualitative data for this participant.

#### Data analysis

Qualitative data analysis followed Braun and Clarke’s six steps for thematic analysis [35], with recommendations from Roberts [44] for deductive/inductive code development and Nowell [45] for enhanced rigour. As our analytical interests and motivation for conducting interviews were intended to improve understanding around two main domains, the impact of the SR24/7 program and the role of surfer as bystander rescuers, the analysis adopted a primarily theoretical approach [35]. However, we wanted to allow for the inductive development of unexpected themes from the data, and as such, engaged in an iterative code development process to fully explore and consider elements beyond our original analysist driven intents [44].

WK became familiar with the data as the interviewer, via transcription review and editing, writing and reviewing fieldnotes, and initial readings of all transcripts [35]. WK coded the transcripts using NVivo [Computer Software], giving full and equal attention to all aspects of the data with special consideration for non-dominant narratives [35]. WK maintained a reflexive journal to track developing thinking around additional inductive codes and chart ideas around emerging themes; the author team met regularly for debrief meetings throughout the coding process [45]. Searching for, reviewing, defining, and naming themes [35] was an iterative process, the author team documented and fully discussed discrepancies and differing interpretations of the data before arriving at consensus for final themes and representative quotes.

### Part three: pre-post test

Surfing Victoria collects routine pre- and post-program surveys for quality improvement purposes for each SR24/7 course, Part Three comprises an analysis of this data subset from Victoria. At the time of this evaluation, NSW and NZ did not collect these data.

#### Participants and data collection

Surfing Victoria requires each course participant to complete an enrolment form to participate in the program, which asks basic demographic questions (i.e., date of birth, gender) and two baseline confidence questions: “*On a scale of 1–5 how confident would you be of performing a rescue in the surf?*”; and, “*On a scale of 1–5 how confident would you be in performing Cardio Pulmonary Resuscitation (CPR) on someone?*”, where the potential response options were: “1—Not Very Confident”, “2—Not Confident”, “3—Neither Confident nor Not Confident”, “4 – Confident”, and “5—Very Confident.” After the SR24/7 course, each participant receives a follow up survey via email which asks the same two confidence questions about performing a rescue in the surf and CPR on someone else. There is no incentive to complete this follow up survey.

While Surfing Victoria provides the SR24/7 course to individuals under the age of 18 as part of a school program, these responses were excluded from analysis for ethical reasons.

#### Data analysis

Pre-course enrolment forms were linked to post-program surveys via the participant’s email address. Age was calculated based on the date of birth provided by the participant and the date of the SR24/7 course; all participants under 18 were removed and the remaining were grouped into the following age categories: 18–29, 30–39, 40–49, 50–59, and 60 + . We present bar charts for pre- and post-course selections by age and gender [46], and a frequency matrix of pre- and post-course selections to show change between the two surveys.

We evaluated for differences between the pre- and post-survey rescue and CPR confidence questions using paired t-tests, assessing for differences in mean pre- and post-scores overall and within gender categories and age groups. Bonferroni adjusted alpha levels for each test were 0.00625. Our decision to employ and present t-tests here was motivated by a desire to produce results that could be understood quickly by a non-academic audience [47], namely SR24/7 program decision makers and implementers. While there has been some debate around the use of parametric vs non-parametric tests in the analysis of Likert data [48], studies have shown both produce similar results with similar statistical power [49], especially with larger (> 30) sample sizes [48].

### Rigor and reflexivity

In program evaluation, the role of the researchers can influence the evaluation process and outcomes [46]. Data interpretation and translation to findings and conclusions are influenced by researcher positioning including personal characteristics, prior experiences, assumptions, and beliefs [50]. For example, an interview is not a passive, neutral data collection tool: it involves interaction between people to ultimately produces contextually based results [51]. As such, researcher positioning is an inherent part of this study and its knowledge generating process; recognising and addressing the role of the researchers involved serves to increase rigour [52].

WK (male) conducted quantitative analysis for Parts 1 and 3, all interviews for Part 2, and was the primary coder in qualitative data analysis in Parts 1 and 2. WK is a drowning prevention and public health researcher, has professional ocean lifeguard and volunteer surf lifesaving experience, is an active body surfer, and originally from North America. RB (male) is a multidisciplinary beach safety researcher with expertise in surf zone hazards, experienced body surfer, and has previous volunteer lifesaving experience. AP (female) is a public health researcher with expertise in injury and drowning. RB and AP have previously engaged in research related to bystander rescue, and during this study served as PhD Supervisors for WK.

### Ethics and consent

Participants in Part One and Part Two received Participation Information and Consent Statements and provided their consent to participate in the study. Part Three involved secondary data analysis of already existing data as defined by the National Statement on Ethical Conduct in Human Research (NSECHR), Section 3.1.52; a waiver of consent was granted following guidelines from NSECHR Section 2.3.10 [53]. This study was approved by the University of New South Wales Ethics Human Research Ethics Advisory Panel H under study number HC220037.

## Results

### Part one: retrospective survey

**Fig. 2:**
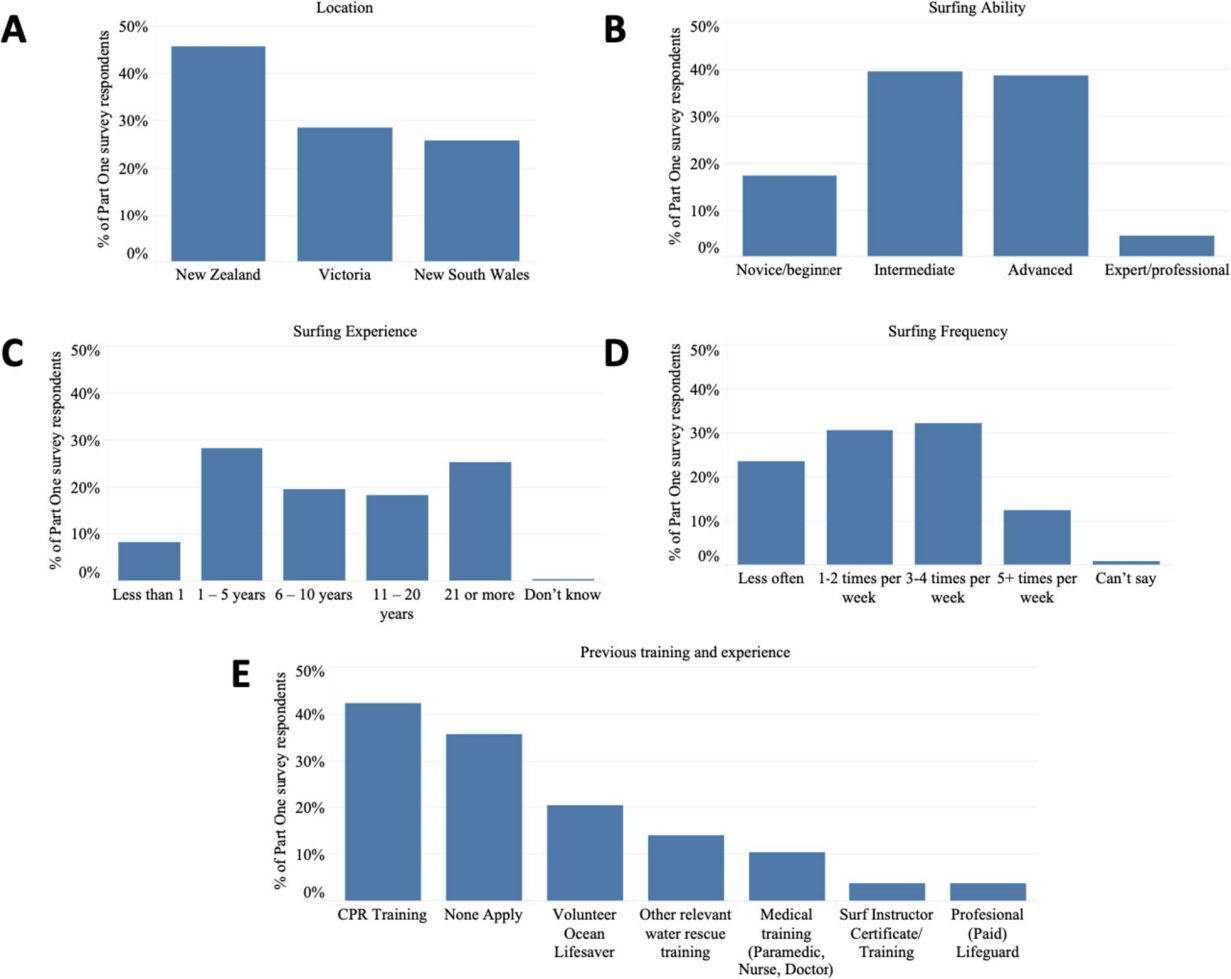
Number of respondents by **A** location, **B** self-reported surfing ability, **C** surfing experience, and **D** surfing frequency at the time of the SR24/7 course, and **E** prior relevant training and experience. Note numbers for prior relevant training and experience add to more than 241 (100%) as respondents were able to choose more than one option

Out of 271 responses, ten were excluded because they did not take the course in the past two years, an additional eight were excluded because they were under the age of 18 years, and twelve completed the screening questions, but did not complete any other question in the survey, leaving 241 responses for analysis. Respondent demographics and background are presented in Fig. 2.

#### Motivation to sign up for the course

Respondents reported finding out about the course in different ways, the most common were from a social media post (*n* = 85; 35.3%), from their board rider club (*n* = 74; 30.7%), and from a friend or family member (*n* = 59, 24.5). In analysis of open text responses to the question “*Why did you sign up to take the Surfers Rescue 24/7 course?”,* several themes were identified that provide insight into the varying motivations to participate.

##### Motivation to participate theme #1: work

Several respondents (*n* = 31; 16.2%) commented that their participation in the SR24/7 course was related to their work at a surf school (e.g., as an instructor), as a surf coach, or other educator role (e.g., an outdoor or physical education teacher):

“I am a teacher that takes students surfing. The chance of needing to execute a rescue is relatively high so thought it would be good to learn best practise.”

(Advanced surfer, 11-20 years surfing, New Zealand)

##### Motivation to participate theme #2: upskilling, learning, improving safety, and building confidence

A third of respondents (*n* = 81; 33.6%) described their motivation for participation in the SR24/7 course was a desire to learn new skills related to surf rescue:

“We have had a lot of drownings in NZ and at my local beach. I wanted to learn rescue techniques to be able to help if I ever am in a rescue situation”

(Intermediate surfer, 6-10 years surfing, New Zealand)

Others (*n* = 39; 16.2%) specifically commented on building their confidence to help in a rescue situation, many also describing a desire to learn how to keep themselves safe while helping others:

“I wanted to learn some skills (which I definitely did!) and feel more confident to help if needed.”

(Intermediate Surfer, 21+ years surfing, New South Wales)

“I had saved a few people in the surf and wanted better methods and to feel safe doing so.”

(Intermediate Surfer, 11-20 years surfing, New South Wales)

##### Motivation to participate theme #3: helping others

Helping others was a motivation to take the course and a common thread in a quarter of the responses (*n* = 63; 26.1%). Twenty-three respondents (9.5%) commented on being able to help friends and other surfers (“So I can rescue my mates in the water if required”; Intermediate Surfer, 1–5 years surfing, New South Wales), 19 (7.9%) mentioned general beachgoers and tourists (“…. tourists regularly visit and are not always familiar with the behaviour of water”; Intermediate surfer, 21 + years surfing, Victoria), and 15 (6.2%) mentioned their children or family members (“Because my children are in the ocean a lot and I am there supervising” Intermediate Surfer, 21 + years surfing, New South Wales). Eight respondents (3.3%) specifically mentioned wanting to help if incidents occurred in remote or isolated locations without lifeguards:

“To help others if something ever went wrong and no lifeguards were present”

(Novice Surfer, <1 year surfing, Victoria)

“I wanted to know how to save someone in the water and share this knowledge with others. I surf isolated waves frequently and I feel the more safety knowledge around this subject, the better.”

(Advanced surfer, 11-20 years surfing, New Zealand)

##### Motivation to participate theme #4: previous incidents

Previous surfer rescues were also a strong motivating factor for people to sign up to the course. Several respondents (*n* = 21; (8.7%) discussed incidents that they themselves had been involved in and a desire to be better prepared if a similar situation happened again:

“Prior to the course I had saved a number of people but did not feel fully equipped to deal with more serious situations.”

(Intermediate Surfer, 11-20 years surfing, New South Wales)

“To be of assistance if people get into trouble while I’m surfing. I had to rescue a mate the year prior and muddled my way through it and so wanted to know what to do if it happened again”

(Intermediate Surfer, 1-5 years surfing, Victoria)

One respondent commented that that they had not personally had to make a rescue, but saw or heard from a friend about a situation where surfers intervened, and wanted to be prepared:

“A friend had to perform a serious rescue (family caught in rip- one guy unconscious) while out surfing and it made me realise that if that had been me I would have had no clue what to do.”

(Intermediate surfer, <1 year surfing, New South Wales)

Even events that the person was not present for nor heard about directly from a friend proved to be a motivator for another participant:

“There had been a boat accident in the media where surfers saved multiple lives, given the time I spend in the water I wanted to know what to do if I came across something similar.”

(Advance surfer, 21+ years surfing, New Zealand)

#### Course impact on the participants

Participants responded positively to the course. The variability of responses to individual questions is presented in Table 1 and composite mean scores, standard deviation, and regression results by surf ability, experience, and frequency are presented in Table 2. While some of the composite mean scores varied at a statistically significant level (Table 2), it is important to note the mean differences between the response categories were minimal – under 0.2 for every group. Overall, surfers with less experience (1–5 years: 5.96 ± 1.08; Less than 1 year: 6.04 ± 0.98; *p* = 0.014; p = 0.014) and lower self-reported surfing ability (Intermediate: 5.94 ± 1.10; Novice/Beginner: 6.01 ± 1.08; *p* = 0.002) had more positive attitudes related to their learning in the course. Course satisfaction was very high (6.59 ± 0.75), and did not differ by ability (*p* = 0.74), experience (*p* = 0.299), or frequency (*p* = 0.831). Surfers with lower self-reported ability (Intermediate: 6.4 ± 0.97; Novice/Beginner: 6.59 ± 0.72; *p* = 0.005), less experience (1–5 years: 6.42 ± 0.91; Less than 1 year: 6.66 ± 0.61; *p* = 0.025), and lower surfing frequency (1–2 times per week: 6.34 ± 0.96; Less often:6.55 ± 0.79; = 0.021) had more positive attitudes about the role of surfers in coastal safety, although it is worth noting that mean score regarding the role of surfers in coastal safety was over 6 (on a scale from 1 to 7) in every category.

**Table 1:** Selected course evaluation questions with frequency responses

	**Missing**	**Terrible**	**Very Poor**	**Poor**	**Neither good nor bad**	**Good**	**Very good**	**Excellent**
Overall, I would rate the Surfers Rescue 24/7 course as:				1 (0.4%)		35 (14.5%)	96 (39.8%)	109 (45.2%)
	**Missing**	**Strongly Disagree**	**Disagree**	**Somewhat Disagree**	**Neither Agree nor Disagree**	**Somewhat Agree**	**Agree**	**Strongly Agree**
I am happy that I participated in the Surfers Rescue 24/7 course		4 (1.7%)				2 (0.8%)	33 (13.7%)	202 (83.8%)
I would recommend other surfers participate in a Surfers Rescue 24/7 course			1 (0.4%)			4 (1.7%)	55 (22.8%)	181 (75.1%)
I learned new skills at the Surfers Rescue 24/7 course						6 (2.5%)	67 (27.8%)	167 (69.3%)
I remember most of what I learned at the Surfers Rescue 24/7 course				2 (0.8%)	6 (2.5%)	45 (18.7%)	130 (53.9%)	58 (24.1%)
Completing the Surfers Rescue 24/7 course improved by ability to identify ocean/beach hazards and hazardous surf conditions	2 (0.8%)	3 (1.2%)	27 (11.2%)	11 (4.6%)	43 (17.8%)	48 (19.9%)	66 (27.4%)	41 (17%)
Completing the Surfers Rescue 24/7 course improved by ability to recognize a swimmer or surfer in distress	2 (0.8%)	3 (1.2%)	15 (6.2%)	5 (2.1%)	25 (10.4%)	52 (21.6%)	93 (38.6%)	46 (19.1%)
Completing the Surfers Rescue 24/7 course improved my ability to safely rescue someone who needs help in the surf	4 (1.7%)	2 (0.8%)	1 (0.4%)		5 (2.1%)	25 (10.4%)	101 (41.9%)	103 (42.7%)
As a surfer, I have a responsibility to look after the safety of others (both surfers and swimmers) in the water when I am surfing	4 (1.7%)		4 (1.7%)	4 (1.7%)	15 (6.2%)	40 (16.6%)	83 (34.4%)	91 (37.8%)
I believe that all surfers should complete a basic lifesaving and CPR course	4 (1.7%)			3 (1.2%)	8 (3.3%)	20 (8.3%)	58 (24.1%)	147 (61%)
I believe that the Surfers Rescue 24/7 course is an important initiative and it provides value to the community	6 (2.5%)					7 (2.9%)	47 (19.5%)	181 (75.1%)
I have used what I learned from the Surfers Rescue 24/7 course in real life situations		12 (5%)	40 (16.6%)	10 (4.2%)	78 (32.4%)	41 (17%)	31 (12.9%)	29 (12%)
Today, I could rescue someone who needs help in the surf and keep myself safe while doing it	4 (1.7%)	2 (0.8%)		2 (0.8%)	5 (2.1%)	47 (19.5%)	102 (42.3%)	79 (32.8%)
	**Missing**		**Less often than before I completed the course**		**About the same as before I completed the course**		**More often since I completed the course**	
In comparison to the time before you completed the Surfers Rescue 24/7 course, how often do you verbally warn swimmers or other surfers about ocean hazards or dangerous situations when you are surfing? For example, tell swimmers about a rip current or submerged rocks	6 (2.5%)		2 (0.8%)		136 (64.7%)		77 (32.0%)	
	**Missing**		**Less aware than before I completed the course**		**About the same as before I completed the course**		**More aware since I completed the course**	
Since completing the Surfers Rescue 24/7 course, has your awareness of others who might be in trouble in the surf and on the beach changed?	6 (2.5%)		1 (0.4%)		84 (34.9%)		150 (62.2%)	
	**Missing**		**No, my opinion is the same as before the course**		**Yes, my opinion changed**			
Did the Surfers Rescue 24/7 course change your opinion on the importance of surfers receiving basic rescue and/or CPR training?	4 (1.7%)		118 (49.0%)		119 (49.4%)

**Table 2:** Composite mean score, standard deviation, and regression results by surf ability, experience, and frequency

Composite Domain	Composite Score		Linear Regression Results			
**Variable**	**Mean**	**Sd**	**Estimate (95%CI)**	**St. Error**	**T Value**	***P* value**
**Course Learning**						
Total	5.84	1.28	~			
Surf Ability						
Expert/Professional	5.50	1.77	-0.17 (-0.27—-0.06)	0.05	-3.13	0.002*
Advanced	5.71	1.43				
Intermediate	5.94	1.10				
Novice/beginner	6.01	1.08				
Surf Experience						
21 years or more	5.70	1.50	-0.08 (-0.15 -0.02)	0.03	-2.47	0.014*
11 – 20 years	5.80	1.37				
6 – 10 years	5.80	1.22				
1 – 5 years	5.96	1.08				
Less than 1 year	6.04	0.98				
Surf Frequency						
5 + times per week	5.75	1.47	-0.08 (-0.17—0.01)	0.05	-1.69	0.093
3–4 times per week	5.75	1.42				
1–2 times per week	5.93	1.15				
Less Often	5.93	1.06				
**Course Satisfaction**						
Total	6.59	0.75	~			
Surf Ability						
Expert/Professional	6.67	0.54	-0.01 (-0.09—0.07)	0.04	-0.33	0.74
Advanced	6.58	0.69				
Intermediate	6.55	0.83				
Novice/beginner	6.66	0.75				
Surf Experience						
21 years or more	6.57	0.82	-0.03 (-0.08—0.03)	0.03	-1.04	0.299
11 – 20 years	6.55	0.77				
6 – 10 years	6.54	0.79				
1 – 5 years	6.63	0.71				
Less than 1 year	6.68	0.57				
Surf Frequency						
5 + times per week	6.58	0.83	-0.01 (-0.07—0.06)	0.03	-0.21	0.831
3–4 times per week	6.60	0.71				
1–2 times per week	6.54	0.89				
Less Often	6.63	0.57				
**Role of Surfers in Coastal Safety**						
Total	6.37	0.95	~			
Surf Ability						
Expert/Professional	6.17	1.02				
Advanced	6.27	0.99	-0.15 (-0.25—-0.05)	0.05	-2.83	0.005*
Intermediate	6.40	0.97				
Novice/beginner	6.59	0.72				
Surf Experience						
21 years or more	6.24	1.10	-0.07 (-0.13—- 0.01)	0.03	-2.26	0.025*
11 – 20 years	6.38	0.84				
6 – 10 years	6.36	0.97				
1 – 5 years	6.42	0.91				
Less than 1 year	6.66	0.61				
Surf Frequency						
5 + times per week	6.21	1.11	-0.10 (-0.19—-0.02)	0.04	-2.33	0.021*
3–4 times per week	6.33	0.96				
1–2 times per week	6.34	0.96				
Less Often	6.55	0.79				

^*^Excludes Surfing Experience “Do’t Know”; Surfing Frequency “Can’t Say”

All but six respondents (97.5%) indicated that they would be interested in some sort of related follow-up activity or training: 51.1% (*n* = 123) said they would be interested in an “advanced” SR24/7 course that covered different or difficult rescue situations, 41.5% (*n* = 100) said they would be interested in attending another SR24/7 course to refresh and practice skills, 22.4% (*n* = 54) said they would like to be included in a community call-out team to respond to ocean emergencies in their local area, and 14.9% (*n* = 35) said they would be interested in training to be a lifeguard/lifesaver level of surf rescue, but only 25% of these (3.7% of total; *n* = 9) said they would be interested in becoming a patrolling lifeguard/lifesaver (paid or volunteer).

#### Using skills and knowledge learned from the course

One-hundred and one (41.9%) respondents said they somewhat agreed, agreed, or strongly agreed that they had used skills learned in the course (Table 1). An optional open text response asked participants how they had used their skills. Most respondents (*n* = 155; 64.3%) somewhat agreed, agreed, or strongly agreed that the course had improved their ability to identify ocean/beach hazards (Table 1), one respondent commenting that the course helped them “understand waves and tides better” (Novice surfer, 1–5 years surfing, Victoria), and several others mentioned spotting and navigating rip currents.

Most respondents (*n* = 150; 62.2%) reported that after the course they were more aware of their surroundings and others who might be in trouble (Table 1). An advanced surfer from New South Wales with more than 21 years surfing experience commented that they were “more observant and conscious of [their] surrounding and fellow surfers and ocean users.” Other respondents said that they had a “more heightened awareness of other swimmers in the ocean” (Intermediate surfer, 1–5 years surfing, Victoria) and “used these skills to take a more proactive approach” (Intermediate surfer, 6–10 years surfing, Victoria).

For several, the skills learned in the course translated to action. Nearly one third (*n* = 77, 32.0%) of the respondents reported that they more frequently warned other about hazards or dangerous situations compared to before the course (Table 1). One intermediate surfer with more than 21 years surfing experience from Victoria reported using skills and knowledge taught in the course when they “provided advice to swimmers unfamiliar with beach conditions.”

Thirty-nine respondents (16.2%) reported having rescued someone from the ocean *after* their Surfers Rescue 24/7 course. Respondents reported rescuing a total of 64 people: 53.8% (*n* = 21) rescued one person, 35.9% (*n* = 14) rescued two people, 2.6% (*n* = 1) reported rescuing three people, and 7.7% (*n* = 3) reported rescuing four people. Of these, the respondents estimated that 26 (40.6%) would have drowned had they not intervened.

One response to the question “How have you used the skills or knowledge you learned from Surfers Rescue 24/7” is worth exploring further. An intermediate surfer with 1–5 years surfing experience from Victoria said:

“I witnessed a person stuck in a rip, whilst I did not conduct the rescue myself, I urged others around me who were more confident to do so.”

This response illustrates that the respondent knew their limits and avoided a rescue they may not have been equipped to handle. While it is unknown how other course participants would respond in a similar situation, it is encouraging that 85.5% (*n* = 206) of all respondents chose “ensuring your own safety and assessing conditions” as the most important component of a surfer’s first response in a rescue situation, and separately, 84.2% (*n* = 203) correctly identified that a surfer should place their board between themselves (the rescuer) and the victim; a course-taught technique to maximise safety of the surfer-rescuer. The quote above also illustrates that increased awareness and improved ability to spot both rips and people in trouble is valuable, even for those who are less confident to perform a rescue. This person kept themselves safe, but encouraged others who were more confident to act.

### Part two: interviews

A total of 14 individuals were recruited from the Part One Retrospective Survey and participated in an in-depth interview (Table 3). Results from qualitative analysis of the interview transcripts are presented below as themes (Table 4) which further characterise the role of surfers as bystander rescuers, provide new understanding of how the SR24/7 course influences surfers, and identified ways that the program can improve in the future.

**Table 3:** Part Two In-depth interview participant characteristics

Interview Participant #	Location	Gender	Age	Surf Experience	Surf Ability
1	New Zealand	Female	41	11 – 20 years	Advanced
2	New Zealand	Female	38	1 – 5 years	Intermediate
3	New Zealand	Female	22	1 – 5 years	Intermediate
4	Australia—NSW	Male	66	21 or more	Advanced
5	New Zealand	Male	40	21 or more	Advanced
6	New Zealand	Female	29	6 – 10 years	Advanced
7	Australia—NSW	Male	49	21 or more	Advanced
8	New Zealand	Male	31	1 – 5 years	Advanced
9	Australia—NSW	Female	38	11 – 20 years	Advanced
10	New Zealand	Female	41	11 – 20 years	Advanced
11	Australia—NSW	Male	52	6 – 10 years	Intermediate
12	Australia—VIC	Male	39	11 – 20 years	Advanced
13	Australia—VIC	Female	51	1 – 5 years	Novice
14	Australia—VIC	Male	47	11 – 20 years	Advanced

**Table 4:** Part two interview themes and sub themes

Theme	Title
1	Custodians of the ocean: Personal beliefs about surfers’ role in coastal safety
1A	It’s not all about lifeguards, surfers are filling in the gaps
1B	Acknowledging not everyone wants to be a custodian
2	The danger is real, and the SR24/7 course makes surfers safer
3	It’s not all life and death, sometimes a quick chat can prevent a tragedy
4	SR24/7 improved awareness & confidence
5	When rescues unfold, having a few techniques to draw on helps
6	Practising the rescue techniques is important
7	Other Course Recommendations
7A	Need to cater to surfers of various skill levels and physical strength
7B	Expanded course content
7C	Regular refreshers and/or pathways to further training
7D	Surf and ocean conditions on the day of the course matter
7E	Suggestions for increasing participation and motivating course sign-ups

#### Interview theme 1: custodians of the ocean: personal beliefs about surfers’ role in coastal safety

Participants commented on several factors which position surfers as a de facto component of the coastal safety infrastructure in their communities. In general, interview participants expressed the belief that surfers have a responsibility to ensure the safety of others. One participant described the identify of a surfer as being a “custodian of the ocean” which, to them, involved keeping the beach clean and beachgoers safe (#12). Another participant described the surf school on their local un-lifeguarded beach as the “drop-off point for people to send their kids”, commenting further that the parents: “knew who we were, and they knew that we were confident in the water and if their kids had issues we would be out there” (#1). Other participants brought up their ocean experience (#4, #6, #7, #8, #12, #13, #14), having a flotation device to help (#5, #6, #7, #13), knowledge of the local area and conditions (#1, #6, #7, #9, #12), and already being out in the water or close to where people needed help (#6, #9, #13, #14):

“I guess if you, if you’ve got a bit of experience, you've already got a flotation device in the water and you’re already next to the person, so you’re almost like, you know, you’re a first responder, right?” (#13).

##### Interview sub-theme 1A: it’s not all about lifeguards, surfers are filling in the gaps

The strongest and most frequent comments on the surfing community’s role in coastal safety, addressed by ten of the 14 interview participants, were the fact that they (surfers) are frequently present in places without lifesaving services:

“We don’t have lifeguards up here, but we have surfers.” (#1)

“We have very few surf [lifesaving] clubs and a lot a lot of coastline where there’s only surfers.”(#4)

Or in places *with* lifesaving services, that surfers were usually around before and after those services were operational:

“Surfers are definitely a big part of the picture because they're there when Surf Life [Saving] isn't.” (#7)

“You know surfers are out there before the flags go up, and there are a lot of surfers out at dusk after the flags go down…” (#11)

Some interview participants (#5, #7, #11, #14) had completed training to be surf lifesavers and were previously or still involved in volunteer lifesaving but clarified that the SR24/7 course was distinct from their previous training. One participant commented that the SR24/7 course added value:

“The difference with this course was that its surfboard based, designed for surfers. So instead of having rescue boards, we had our own surfboards which was really good. The best thing about it was the towing techniques for [surfers].” (#7)

Another interview participant (#5) who is also involved with surf lifesaving acknowledged historical friction between lifeguards and surfers, but presented a hopeful, outside-the-box vision for what community-level coastal safety might look like in the future:

“There has always been that separation between [lifeguards and surfers] …. But it is getting better and better. Essentially, if we can join those two closer, we’re going to have coverage dawn to dusk throughout the day… And we’re going to really make a difference in drowning prevention.” (#5)

##### Interview sub-theme 1B: acknowledging not everyone wants to be a custodian

It’s important to consider that those interviewed might not represent the entire surfing community: they completed a SR24/7 course and had conducted some sort of rescue to even be recruited into the research. When asked if they thought others shared their views on the role of surfers in coastal safety, two participants communicated that most would but acknowledged some might not:

“I feel like a lot of surfers are actually quite genuine and they're gonna lend a helping hand in the water if they need to. But you will still have the handful of like old dudes, who you know, if you look at them the wrong way in the water, they're gonna try and shoo you away and I feel like those guys are just kind of old and stuck in their ways and like, it'd be pretty hard to get them to want to go out of the way to help someone else.” (#8)

“There are some people who really don't give a shit what's going on around them.” (#4)

#### Interview theme 2: the danger is real, and the SR24/7 course makes surfers safer

After completing the course, the surfers interviewed were keenly aware of the risks to their personal safety when assisting others in the surf, ten of the 14 participants echoed that a major takeaway from their SR24/7 experience was the strong emphasis on personal safety. When asked to reflect on their experience *before* the course, some participants (#2, #10, #13, #14) admitted that they probably would not have thought too much about their own safety:

“I probably never would have placed importance on my own well-being in my thought process to help someone. And I think I'm not sure of the word to say that, but [the course] just made me aware that I also have to put myself within the split-second equation… that I need to protect myself.” (#2)

The course’s focus on keeping yourself safe in rescue situations is well justified, multiple respondents (#1, #2, #3, #6, #8) shared reflections on dangerous rescue situations they had personally been involved with or had heard about from fellow surfers. One participant (#2) recounted a rescue situation which served as a motivator to enrol in the SR24/7 course. While surfing, the participant noticed a swimmer had been taken away from the beach by a large rip current. Paddling over, she only saw the hair of the victim on the top of the water as they struggled to keep their head above water:

“…and so, I like dove in, and I pushed them up, but when I pushed them out, I realized it was like a hundred and something kilo person. And then like, and as he was coming up, he kept pushing me under the water. I tried just holding him [up], but he kept pushing me under the water and then I tried to get away.” (#2)

The participant eventually got away from the victim, got them to calm down and hold onto the surfboard, but was then faced with getting the person back to shore in dangerous surf conditions as it was getting dark. Another surfer came out to help and it took “about 20 to 25 min” to get back to shore. In reflection, participant #2 commented:

“I was pretty petrified… I think it was terrifying just because I realized like we could both drown. I put other people at risk to try and help.” (#2)

The course’s influence on safety thinking and actions was apparent (“The main theme that came after completing the course was maintaining your own safety within the water despite what's going on.” #6), other participants (#1, #9, #10, #11, #13) commented that they had applied the safety lessons and techniques from the course, some (#3, #13) specifically discussed the course-taught technique to always place your board in between yourself and the victim:

“… The first thing that I thought of when I was out there was Oh, that's right, they said, if I approach them I've got to put the board between me and the person. So that's when I actually did.” (#13)

Others (#3, #5, #6, #7, #8) commented on assessing their own abilities to help in various situations, being realistic with themselves about their own skill level and the conditions in order to not put themselves at risk:

“Someone got um, pulled out along the side of the rock wall in a rip and got straight out in the danger zone, they probably would have drowned. I got up and I was thinking about getting in, and another guy came down and I said ‘Can you get out?’ So yes, I gave him my board and he went instead… I didn't actually feel fit enough myself to do it.” (#7)

#### Interview theme 3: it’s not all life and death, sometimes a quick check-in can prevent a tragedy

While participants did describe critical rescue scenarios (Theme 2), they also conveyed that surfers are involved in a range of other prevention/safety related interventions that are not as serious or dangerous:

“I usually just paddle with someone in, like to make them feel comfortable” (#2)

“I just felt like she was too far out and that's why I paddled over. I was like, Oh, do you want to just rest on my board for a second?” (#9)

Some participants (#2, #8, #11) expressed that they feel comfortable speaking up and warning people at the beach about dangerous conditions:

“I see lots of families like, you know, way down from the flags or whatever just swimming and kind of in gnarly spot. I don't want to, you know, cause any controversy, I'm just kind of looking out for them. I'm like, ‘look, this isn't a great spot to be swimming. You should go down 100 meters and go swimming between the flags because no one's really looking out for you here.’” (#8)

Others conveyed that they didn’t feel it was their role to speak up. One participant recalled an event where he did not want to say anything, and the people ended up needing rescue:

“I saw two guys on like learner boards or something and I was like, I don't think they should be paddling out, but I didn't want to be that know-it-all wanker and so I didn't say anything. And then one of them ends up getting rescued, like he got swept down then caught like at the end [of the beach] where it's sheer cliff and he got like marooned there and had to get the SES [State Emergency Services] and bloody helicopters and stuff. And I was like, I bet that's one of those guys that I should have told an hour ago not to paddle out.” #12

One concern about verbally warning people or speaking to the public was how they would react. While “most people take it pretty well” (#8), one participant expressed: “I’ve never had a positive experience doing that even as I’ve tried to be as calm and like as educational as possible” (#12). One suggestion for future SR24/7 courses was to include some tips and suggestions for talking to strangers, tourists, or other surfers about safety situations.

#### Interview theme 4: SR24/7 improved awareness & confidence

Increased awareness of one’s surroundings in the water while surfing and higher levels of self-efficacy to assist in unsafe situations were reoccurring themes in the interviews. Several participants (#4, #5, #8, #9, #11, #12) communicated that after the course, they were more conscious of what swimmers and other surfers were doing and how the conditions were affecting them and everyone else.

“I’m certainly more aware of what’s going on. I take more notice of you know, other people. I’m a bit more aware of it all.” (#4)

For several of the participants, this increased awareness and vigilance was also associated with improved confidence in their capacity to help in various coastal safety situations, which was mentioned by 11 of the 14 interview participants.

“I feel like I have my head on a swivel more because I feel like now I am a bit more confident to help out people if they need it. I'm always looking around for sure.” (#8)

The improved confidence expressed by participants was multilayered. First, surfers felt they had learned the skills and techniques at the course to intervene in a drowning situation and were now more comfortable in doing so.

“I probably feel a bit more confident that I’d be able to put someone on my board and be able to get them in.” (#10)

However, participants also expressed high levels of self confidence in their own ocean knowledge and experience. From the rescue situations described by participants, it was apparent that that they 1) were aware of what was happening around them, 2) realised something about the situation didn’t look/feel right or had the potential to turn into something worse, and 3) decided to act on that information by checking in on the person. In short, they trusted themselves and felt empowered to help, with some participants clearly communicating this improved confidence was directly from the SR24/7 course.

“Yeah, like the time when I did a rescue, the girl that I found that was in trouble. Before the course I probably would have been like, oh, she’s alright. But after the course, I was more aware of it and was like, I’m just gonna go over and see how you are just in case.” (#9)

**“**It's a matter of being aware of your surrounds. And you know that I've made, oh, probably at least half a dozen, and I don't specifically call them rescues, but know you just, you just watch, you know, these are just swimmers that get a little bit out of their depth, they don’t look right and, and you go to them, you know… the key to me is to get to them before they start to panic… just paddle over, just say ‘hey, mate, you know, having trouble touching bottom there?’ you know, just check on them.” (#11)

#### Interview theme 5: when rescues unfold, having a few techniques to draw on helps

Several common threads emerged from recounting of the participant’s rescue stories which highlighted the importance of the SR24/7 course. In serious situations, when someone was drowning or conditions were particularly challenging, things developed quickly and could be a bit chaotic. In these moments, the surfers had to account for several different factors in a “split-second equation” (#2), making “value judgements” (#7) about what to do.

“I don’t know, like sort of your instinct kicks in and I, like, when things start happening, you just sort of start making decisions quite quickly.” (#1)

Across the interviews, three input factors emerged as being important in these moments of high stress and fast decision-making:

1. The surfer’s ocean experience and skills: Physical fitness, ability to operate/navigate waves and ocean currents, comfort in the surf.
2. The surfer’s local knowledge about that specific beach: the location of rip currents, rocks, entry/exit points, sand bars, etc.; how surf and water move in that location.
3. The surfer’s previous training and knowledge of rescue techniques.

As described in Theme 4, the course served to empower and improve confidence in relying on their ocean experience and local knowledge with strong consideration for personal safety, but most tangibly, the course provided participants with several basic rescue techniques designed specifically for surfers. In conversation about major takeaways from the course, in addition to personal safety previously discussed in Theme 2, 12 of the 14 participants discussed the rescue and towing techniques they were taught:

“I guess the really useful and important part for me was, well, learning how to like pull someone out and put them on your surfboard and bring them back into shore safely.” (#3)

“The [rescue] techniques we were showed were great… I learned loads of new stuff.” (#11)

Not knowing what to do or considering using their boards in the ways shown in the course was a common theme:

“One thing was the how to use a surfboard in a rescue … like the little techniques of having the board like side by side and kicking in together or just, like, I would never really thought of that.” (#9)

“Well, at the start I wouldn’t have had a clue how to roll over a person [onto the board] … what a real struggle that probably would have been. But no, I found that technique was really good.” (#4)

Two participants (#5, #6) recounted that their SR24/7 instructors ran a facilitated debrief session at the conclusion of their course, which they found “really helpful” (#6). In these discussions, course participants were invited to share a rescue situation were involved in, and then, with guidance from the course instructors, the individual involved, and the rest of the group then collectively identified what went well, and what could have been improved. Hearing examples of several different types of rescues, and then exploring multiple potential solutions provided participants with a variety of contexts that they felt could be helpful in future rescue situations.

#### Interview theme 6: practising the rescue techniques is important

The in-water practical part of the course where the participants learned and tried the different rescue techniques was extremely valuable to the participants (“It was so helpful to practice it in the water” #6). Participants discussed this part of the course multiple times in their interviews, helping to provide some information on why they thought it was so valuable. For some (#2, #2, #3, #5, #6, #8, #11), it was a surprisingly difficult task:

“I think that’s probably what was best, the practice on the beach. It was like pretty nuts but when we did it, and how we did it was really, really cool. Like, it wasn't protected too much like they kind of put you out there and let you struggle with it. I really liked that. There was nothing easy about it.” (#2)

“I was actually really knackered, like, I'm quite a fit person but it actually took a lot out of me doing a rescue.” (#1)

However, others (#8, #10) commented that rescuing others with the course-taught techniques was easier than they expected:

“I think going out and doing it in the actual water was great but wasn't as hard as what it actually looked like on the theory.” (#10)

Following up on why it was easier than expected, being able to try out different methods with their fellow participants and instructress and figure out what worked for them was identified as a positive aspect of the in-water session:

“I think because there’s a couple of different techniques. You sort of tried each one. I can’t remember if it was two or three, but we sort of found the one that suited us better, like with how to pull someone onto your board and like that. Yeah. So I think they look difficult and especially when you’re trying to pull someone who is quite heavy and big or unconscious that can’t really help you. It was a bit like, Oh, I don’t think I can do that. But then giving it a go out in the water was quite helpful.” (#10)

#### Interview theme 7: course recommendations

Overall, participants were extremely pleased with the course (“It was super awesome” #8), in line with results from Part 1 of this study. They were pleased they had participated and some (#3, #10) recommended it to their friends. The following sub-themes outline both positive elements of the course that would be valuable to continue incorporating or standardise, and a few recommendations for improvement not mentioned elsewhere in this section.

##### Interview sub-theme 7A: need to cater to surfers of various skill levels and physical strength

The other surfers participating in the course represented a “broad spectrum of people [with] a lot of different abilities” (#2) and the course was accommodating of different skill levels:

“It’s a good variation catered for different sizes, different strengths, and different size of boards that you that you’re using in the water. So I think overall everyone took away some good techniques they might have not mastered them all.” (#5)

Others pointed out that some of the rescue techniques seemed like they were “for your average dude” and not built for “beginner women who don’t have a lot of upper body strength” (#2). From these conversations, participants recommended that course instructors continue refining how it can be customized to different skill levels, potentially by ensuring there is a higher ratio of instructors to participants for each course to accommodate smaller groups based on skill abilities. Participants also strongly recommended that the course focus on teaching the rescue techniques to participants using the crafts they normally ride (“I think it would be helpful if people just brought whatever craft they use in the water… so that they can practice what they would use in real life” #14). Another recommendation from some of the female participants was for the course designers to re-visit some of the rescue techniques with the goal of innovating new, or coming up with variations on existing manoeuvres, specifically designed for smaller female surfers.

##### Interview sub-theme 7B: expanded course content

Some SR24/7 courses included a full CPR component, others did not for logistical or other reasons. When asked what recommendations the participants had for the course, CPR and resuscitation training was a common theme.

On recommendations for the course: “Including a bit of CPR and first aid and to that would’ve been really good.” (#10)

Other course content recommendations included: Introductory training on major bleeding control to respond to a shark encounter or laceration caused by a surfboard fin (“fin chop”); Techniques or advice on how to approach a rescue situation if the surfer didn’t have a surfboard; many of the participants in the course were surf instructors who are frequently in the water *without* their board while providing lessons; and an intentional discussion around emergency preparedness and planning, especially for those surfing in remote areas: e.g., where the nearest defibrillator/first aid kit is, mobile phone reception dead zones, access and extraction points in case someone is immobile.

##### Interview sub-theme 7C: regular refreshers and/or pathways to further training

Some participants (#1, #5, #12) commented that they thought would like to receive an update and other opportunities to practice the rescue techniques:

“I just think that maybe it’s something that should be refreshed every two years just like your first aid, you know?” (#1)

Another recommended was that after the course, participants be provided with information on further training opportunities such as the “next level of CPR training… and options to move on to become a qualified surf lifesaver.” (#14).

##### Interview sub-theme 7D: surf and ocean conditions on the day of the course matter

While not necessarily in control of the organisers, it is important to note that conditions on the day of the course have an impact on the ability of the course to run and the experience of the participants. One participant explained that the surf was too big and stormy, so the group did not get much practice in the water:

On recommendations for the course: “I guess the only thing would be that being that, I think the practical applying is so key that if the conditions aren't suitable to get in the water, there have a plan B, like the ocean baths or something, so you can actually definitely get into the techniques.” (#9)

Conversely, another participant explained their course was conducted on a completely flat day, no surf, at a beach that was quite shallow:

“It was just difficult to actually learn the proper techniques in those conditions… and I know we’re not going to have perfect conditions every time, but it was shallow you know it was easy to stand up … so the conditions weren’t real and conducive.” (#11)

Finally, some participants commented that if the conditions were good for surfing on a weekend day, it was unlikely that people would show up for a free course they didn’t pay for, even if they signed up indicating that they would be there, because they would be surfing.

##### Interview sub-theme 7E: suggestions for increasing participation and motivating course sign-ups

Another specific line of inquiry in the interviews sought to understand motivations for signing up for the course and new ways or recommendations to expand access to other parts of the surfing population. Results align well with those derived from the Part 1 retrospective survey, primary motivations for signing up for the SR24/7 course included wanting to improve personal skills and abilities, a desire to keep oneself safe, a rescue event or ocean safety situation that the participant was involved in directly or heard about from a friend or fellow surfer, and being part of a surf community or board riders club.

“I had been in incidents where I'd kind of tried to float someone in, with my board and stuff like that. And then I was like, Oh there's actually like techniques and stuff for how to do it better. So, I thought that would be really helpful for me, for the next time it came around.” (#12)

Recommendations for getting other surfers to sign up to the course include continuing to collaborate with board rider clubs, surf clubs, and local schools; highlighting real life examples of surfers making rescues and hosting courses in locations after a well-publicised incident; partnering with surf industry and retailers to offer discounts on boards, wetsuits, or other gear for people who complete a course; and continuing to offer all female courses.

“I surf with a bunch of students… none of us have booties or anything, because we can’t afford it. If there was like, 20% off booties if you take this course, you know… all of them would do it.” (#3)

“I feel that if you opened the course up to both men and women, I just don’t think you would have got the same numbers of women to sign up.” (#6)

### Part three: pre-post test

**Fig. 3:**
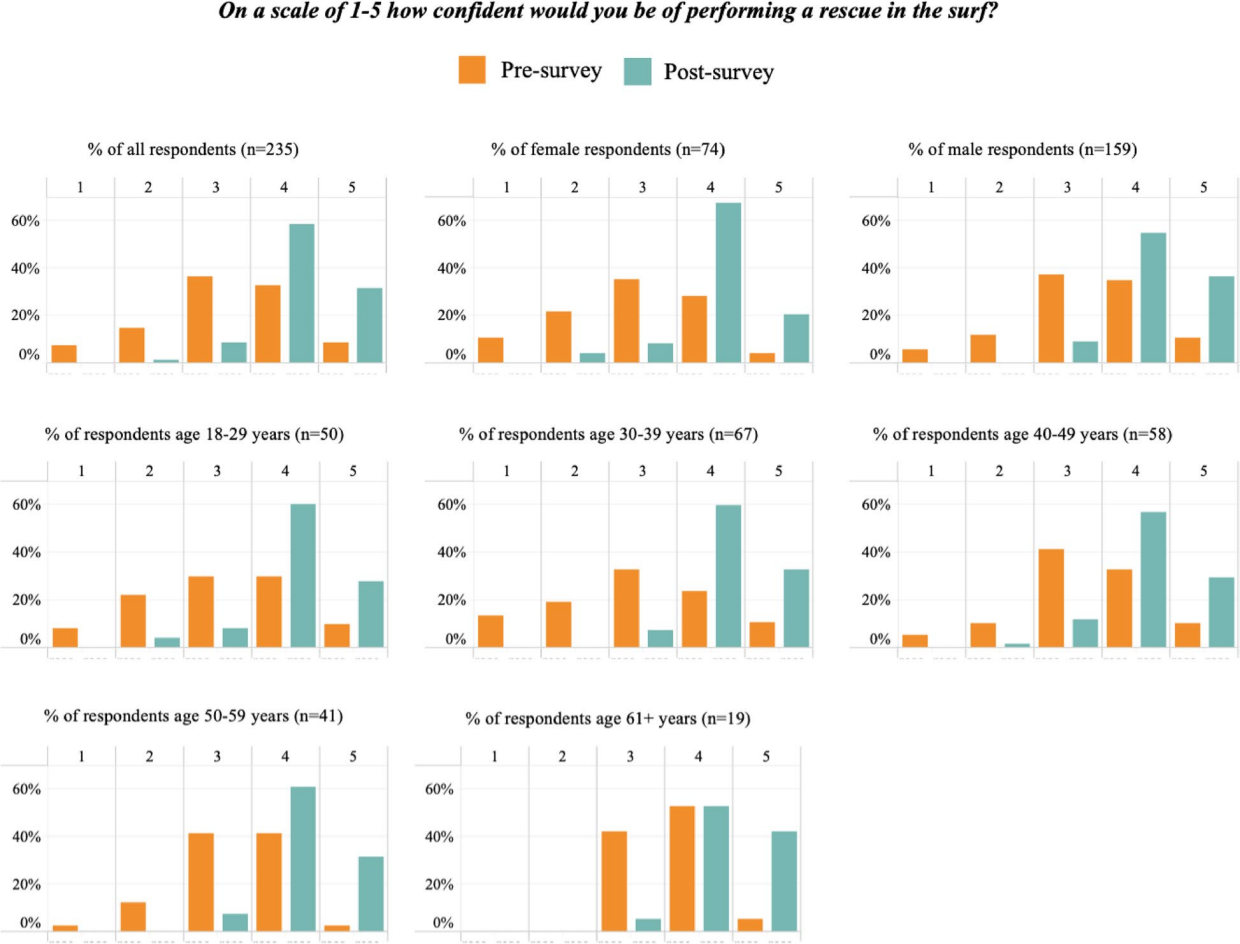
Pre- and Post- course selections for confidence in performing a rescue by gender and age. *Gender data missing for 2 cases

**Fig. 4:**
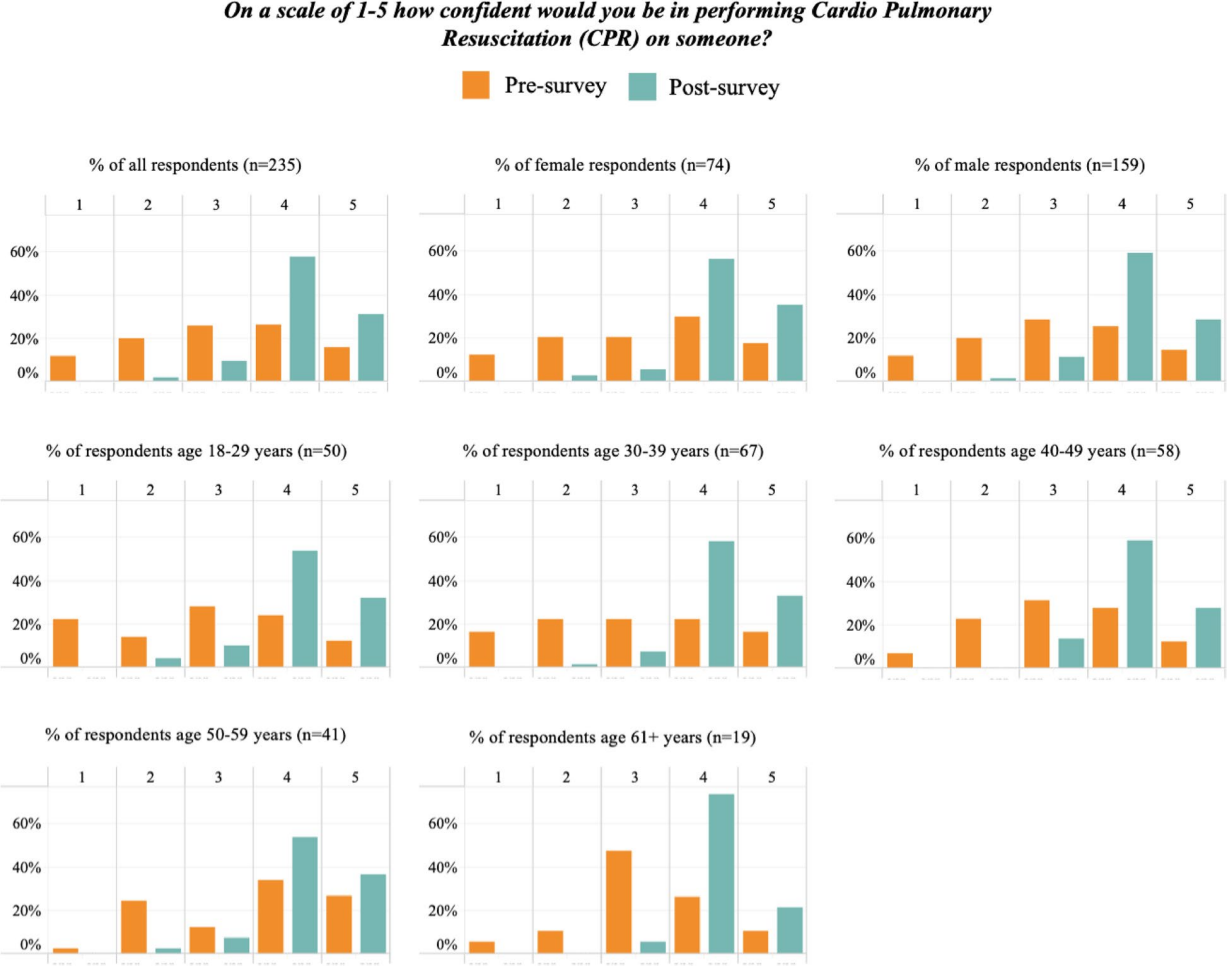
Pre- and post- course selections for confidence in performing a CPR by gender and age. * Gender data missing for 2 cases

A total of 235 pre-post course records from Surfing Victoria were able to be matched for analysis. Confidence scores for the entire cohort improved at statistically significant levels for both rescue and CPR, as did all scores within gender and age categories except for those aged 60 and over for performing a rescue (Table 5). Pre- and Post-course selections for confidence in performing a rescue and confidence in performing CPR are presented by gender and age, in Figs. 3 and 4. Tables 6 and 7 show a breakdown in confidence level change from pre- to post-course for rescues and CPR, respectively.

**Table 5:** Pre and Post mean confidence scores and paired t-test results for performing a rescue or CPR

Variable	Pre-score mean (SD)	Post-score mean (SD)	Mean Difference	*t*	*df*	*p*	*Cohen’s d*
Rescue							
All	3.2 (1.03)	4.2 (0.64)	1 (0.87–1.13)	15.53	234	< 0.001*	1.16
Male	3.33 (1.01)	4.28 (0.62)	0.95 (0.8–1.1)	12.47	158	< 0.001*	1.14
Female	2.93 (1.05)	4.04 (0.67)	1.11 (0.86–1.35)	9.04	73	< 0.001*	1.26
Age 18–29	3.12 (1.12)	4.12 (0.72)	1 (0.71–1.29)	6.86	49	< 0.001*	1.06
Age 30–39	2.99 (1.19)	4.25 (0.59)	1.27 (1–1.53)	9.60	66	< 0.001*	1.36
Age 40–49	3.33 (0.98)	4.14 (0.69)	0.81 (0.56–1.06)	6.41	57	< 0.001*	0.96
Age 50–59	3.29 (0.81)	4.24 (0.58)	0.95 (0.7–1.21)	7.57	40	< 0.001*	1.34
Age 60 +	3.63 (0.6)	4.37 (0.6)	0.74 (0.35–1.13)	3.99	18	0.007	1.23
CPR							
All	3.14 (1.25)	4.18 (0.66)	1.04 (0.91–1.18)	15.06	234	< 0.001*	1.04
Male	3.1 (1.23)	4.24 (0.68)	1.04 (0.89–1.2)	12.97	158	< 0.001*	1.06
Female	3.2 (1.29)	4.14 (0.65)	1.04 (0.77–1.31)	7.69	73	< 0.001*	1.01
Age 18–29	2.9 (1.33)	4.14 (0.76)	1.24 (0.88–1.6)	6.90	49	< 0.001*	1.15
Age 30–39	3 (1.34)	4.22 (0.65)	1.22 (0.96–1.49)	9.12	66	< 0.001*	1.17
Age 40–49	3.16 (1.12)	4.14 (0.63)	0.98 (0.74–1.23)	8.07	57	< 0.001*	1.08
Age 50–59	3.59 (1.2)	4.24 (0.7)	0.66 (0.37–0.95)	4.63	40	< 0.001*	0.67
Age 60 +	3.26 (0.99)	4.16 (0.5)	0.89 (0.5–1.28)	4.82	18	0.001*	1.14

^*^Statistically significant at 0.00625

**Table 6:**
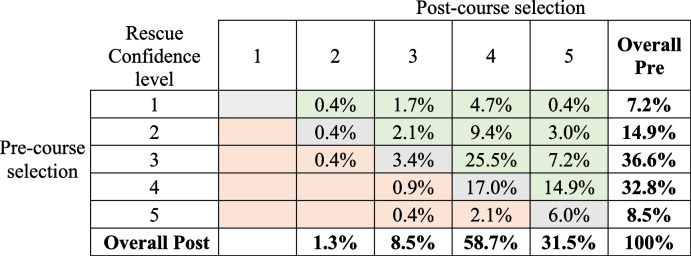
Percent of Part Three respondent’s (*n* = 235) indicated confidence levels for performing a rescue pre- and post-course

^*^Green = Improvement; Grey = no change; Orange = decrease

**Table 7:**
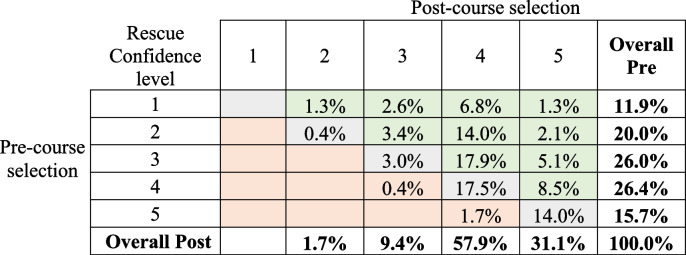
Percent of Part Three respondent’s (*n* = 235) indicated confidence levels for performing a CPR pre- and post-course

^*^ Green = Improvement; Grey = no change; Orange = decrease

## Discussion

This multi-part mixed methods study aimed to fill a gap in coastal safety literature by evaluating a drowning prevention program that teaches surfers skills to prevent and respond to emergency situations in the water. Programs of similar nature have become popular globally, and while the rationale for the intervention is rooted in evidence showing that surfers are already making a significant number of rescues [20, 23, 25, 26] and represent part of the population which has the requisite skillset to do so safely [14], the nature of these courses and their effect on participants has not been documented. To this end, quantitative and qualitative results from this study have 1) offered new insight into the role that surfers play in coastal safety; 2) provided evidence that the SR24/7 course is effective in improving knowledge, awareness, and confidence with a strong emphasis on personal safety; 3) defined strong elements of the course that should continue and be considered best practice for basic rescue training programs for surfers; and 4) identified some areas for improvement and innovation in the program, its recruitment, and follow-up.

Coastal drowning is a complex issue [8] and death rates in Australia are not decreasing despite significant attention and investment [54]. The SR24/7 course in Australia and New Zealand is a coastal safety intervention which is already having a positive impact on the surfing community and preventing drowning death and injury among those who visit beaches, especially unpatrolled locations where most beach drownings occur [55]. If scaled up, this program has the potential to equip and empower thousands of surfers as community coastal safety ambassadors with prevention and rescue capacity, making beaches safer and ultimately reducing the impact of drowning on individuals and society.

SR24/7 course participants gained and retained knowledge and skills from the course, highly valued their experience, and would recommend it to others. One of the most important findings from this evaluation was that the course effectively instilled an ethos of personal safety among the participants, the major concern in any water rescue situation [14] and a historical critique of surfers conducting rescues. Based on results of Part 1 and Part 2 of this study, the course was successful in teaching techniques and empowering surfers to act without increasing their own risk.

This study also shed light on an important aspect of the surfer’s role in beach safety: preventative activities that involve early intervention in the drowning process. The historical focus of surfer involvement in beach safety has been rescue situations where surfers aid swimmers in distress [20, 23, 25, 26]. Results of this study have shown that surfers also frequently check in on swimmers in the water or verbally warn people on the beach about ocean hazards. As ocean lifeguard training programs have encouraged early intervention and preventative actions [56], the SR24/7 course also played an important role in empowering surfers to be more aware of their surroundings and recognize the development of dangerous situations. While some surfers felt comfortable speaking up, one area for improvement in the course would be focused content on conducting preventative check-ins or warnings to people on the beach or in the water.

Intentional engagement with surfers via the SR24/7 course presents an opportunity to dramatically improve community-level capacity to prevent and respond to emergency situations in the surf. Professional lifeguards and/or volunteer surf lifesavers represent the primary coastal drowning prevention effort in Australia and New Zealand and are a frequently recommended coastal safety strategy in the literature [8]. While trained lifeguards and lifesavers will always be a critical component of the coastal safety ecosystem, operations and equipment are expensive (even if volunteer-based), require extensive planning and robust training, and are still limited to specific locations during specific times. Declining volunteerism [57, 58], difficulty hiring and retaining paid lifeguards [59], and budget shortfalls [60] present additional challenges to operating robust ocean lifesaving operations. Of note in this study, 35 participants reported a desire to receive higher level training to a lifeguard/lifesaver qualification level, but only a quarter of those indicated they had interest to work or volunteer as a lifeguard or lifesaver. Further research exploring this discrepancy has implications for the professionalisation of lifeguard organisations and the future of beach safety management systems.

With this evidence showing simple training programs for surfers are effective on multiple levels, expanding training budgets and resources beyond lifeguards and lifesavers to the broader surfer population is worth consideration. In 2022, Surf Life Saving Australia reported just over 48,000 proficient volunteer lifesaver members, and separately, estimated that 1.2 million people surf in the country with 600,000 of those being “frequent surfers” [55]. Providing just one tenth of frequent Australian surfers with basic rescue skills and prevention training would more than double the number of rescuers in the country. Similar conditions exist in New Zealand, with just over 18,000 members [61] and an estimated 315,000 surfers [62]. While surfers don’t “patrol” like lifeguards and lifesavers, they are often in locations where lifeguards and lifesavers are not, and those that complete the SR24/7 course improve their ability to help in times of need and more frequently engage in prevention activities. Augmenting existing coastal safety resources and attention to programs like the SR24/7 course would serve to improve community capacity to respond to and prevent water emergencies.

### Study strengths, limitations, and research challenges

A strength of this study was triangulated learnings from multiple parts, both quantitative and qualitative. Conclusions and results related to surfers’ attitudes and role in coastal safety are subject to selection bias as this was a group who signed up for and completed a basic rescue course, then responded to a request to participate in research about that course. Comparing these results to a broader population of surfers that did not participate in a SR24/7 or similar course would offer valuable insight relevant to the feasibility of scaling the program. This study is also subject to recall bias, although inclusion criteria limited participants to those who completed a course in the past two years, and it was apparent in the interviews that rescue events made a strong impact on the individual. While several steps were taken to increase the rigor of both quantitative and qualitative analysis (“Rigor and reflexivity” section), additional steps would have improved the strength and reliability of results including factorial analysis to evaluate internal consistency of survey items and having a second person fully code each transcript with calculation of Cohen’s kappa to evaluate interrater reliability in qualitative coding. A strength of this study was the inclusion of participants from three locations (NSW, Victoria, and New Zealand).

Evaluating the course faced several logistical challenges. First, COVID-19 lockdowns severely impacted delivery of the course in 2021, restricting the study’s timeframe and recruitment of participants. When courses were able to run, we had originally intended to conduct pre- and post-surveys of program participants, which proved to be challenging. Pre-course registration was mixed, some courses and locations had systems in place, others had less robust methods and always accepted people on the day of which made pre-course data collection difficult and limited. Course organisers expressed concern about presenting participants with lengthy surveys prior to the course, and interview participants affirmed that many surfers would be turned off by additional paperwork. Getting participants to complete timely post-program surveys was also a challenge, text message and email reminders proved ineffective, even with financial incentives on offer. Pre- post-evaluation of knowledge acquisition and rescue technique skills of course participants compared to a control group would be an important extension of this work; future researchers should account for logistical challenges and consider pilot testing their research instruments and processes with a small group of surfers for acceptability.

## Conclusions

This mixed methods study provided key insights into the value and efficacy of the SR24/7 program. Notably, it further characterized the significant role surfers play in coastal safety, verified the program's effectiveness in improving participants' knowledge, awareness, and confidence in rescue and CPR skills, and outlined areas for improvement. Despite persistent challenges in combating coastal drowning rates, interventions such as SR24/7 show promise in not only preparing surfers for rescue situations, but also promoting preventative activities that contribute to early intervention. Emphasizing personal safety, the course successfully equipped surfers with techniques to act responsibly and safely. Expanding such training resources to surfers, an often-overlooked demographic in coastal safety strategies, could substantially enhance community-level capacity to prevent and respond to ocean emergencies.

### Supplementary Information

**Additional file 1. **Part One: SR24/7 past-participant survey questions with theme and composite score, where applicable.

**Additional file 2. **SR24/7 interview guide.

## Data Availability

The datasets generated and/or analysed during the current study are not publicly available due legal and ethical reasons, but deidentified data are available from the corresponding author on reasonable request.
