# Supplementary material for: Impact of a surfer rescue training program in Australia and New Zealand: a mixed methods evaluation
Source: BMC Public Health. 2023 Nov 8;23:2193. doi: 10.1186/s12889-023-17057-w (PMC10631059; doi:10.1186/s12889-023-17057-w)
Supplement: Supplementary file 1 — Additional file 1. Part One: SR24/7 past-participant survey questions with theme and composite score, where applicable. [file 12889_2023_17057_MOESM1_ESM.docx]

**Additional File 1:** Part One: SR24/7 past-participant survey questions with theme and composite score, where applicable

**About Supplementary Material**

Survey questions are provided in **bold text**, with question instructions in *(italics)* if appliable, followed by the possible question responses. Text in brackets […] provides additional information about that question including the theme grouping and if that question was part of a composite score; text in brackets was not part of the original survey and not shown to the participant.

**Survey Questions**

**Are you older than 18 years of age?** *(please select only one answer)*

- Yes
- No (submit and exit survey)

[Theme: Background / Screening]

[Composite Score: Not Applicable]

**Do you provide informed consent to compete this survey?** *(please select only one answer)*

- Yes (click to start survey)
- No (exit survey)

[Theme: Background / Screening]

[Composite Score: Not Applicable]

**Have you completed a Surfers Rescue 24/7 course in the past two years?** *(please select only one answer)*

- Yes, in New South Wales (click to start survey)
- Yes, in New Zealand (click to start survey)
- Yes, in Victoria (click to start survey)
- No (exit survey)

[Theme: Background / Screening]

[Composite Score: Not Applicable]

**1. Please select all the following statements that apply to you *before you completed the Surfers Rescue 24/7 course*:**

I completed training to be a volunteer ocean lifeguard/lifesaver before I took the course

I completed training and worked as a professional (paid) ocean lifeguard before I took the course

I was CPR certified or had completed a CPR training class before I took the course

I was a medical professional (Paramedic, Nurse, Doctor etc.) before I took the course

I had other relevant water rescue training before I took the course

None apply to me

[Theme: Background / Screening]

[Composite Score: Not Applicable]

**2. At the time you took the SR24/7 course, how many years had you been surfing? (*please select only one answer)***

- Less than 1
- 1 – 5 years
- 6 – 10 years
- 11 – 20 years
- 21 or more
- Don’t know

[Theme: Background / Screening]

[Composite Score: Not Applicable]

3. How would you describe your surfing ability at the time you took the course? *(please select only one answer)*

- Novice/beginner (surfing broken waves or unbroken waves straight to the beach)
- Intermediate (surfing unbroken waves left and right)
- Advanced (surfing unbroken waves left and right, turning on the wave, staying close to the breaking part and controlling bottom and top turns)
- Expert/professional (surfing on a higher level than advanced, compete regularly, big wave surfer, have sponsorship [current or previous])

[Theme: Background / Screening]

[Composite Score: Not Applicable]

**4. Around the time you took the SR 24/7 course, how often were surfing? *(please select only one answer)***

- Everyday
- 5-6 times per week
- 3-4 times per week
- 1-2 times per week
- 1-3 times per month
- 3 to 11 times per year
- Less often
- Can’t say

[Theme: Background / Screening]

[Composite Score: Not Applicable]

**5. Around the time you took the SR 24/7 course, how long was a typical session? *(please select only one answer)***

- 15 minutes or less
- 30 minutes
- 1 hour
- 2 hours
- 3 hours
- 4 hours
- 5 hours or more
- Can’t say

[Theme: Background / Screening]

[Composite Score: Not Applicable]

**6a. How did you find out about the Surfers Rescue 24/7 course?**

- Social media post
- News article
- Friend or family
- Board riding club
- Don't remember
- Other:____________

[Theme: Background / Screening]

[Composite Score: Not Applicable]

**6b. Why did you sign up to take the Surfers Rescue 24/7 course?**

__________________________________ [Open text response]

[Theme: Background / Screening]

[Composite Score: Not Applicable]

**7. I am happy that I participated in the Surfers Rescue 24/7 course.**

1. Strongly Disagree
2. Disagree
3. Somewhat Disagree
4. Neither Agree nor Disagree
5. Somewhat Agree
6. Agree
7. Strongly Agree

- Don't know / can't remember

[Theme: Course Satisfaction]

[Composite Score: Satisfaction]

**8. Overall, I would rate the Surfers Rescue 24/7 course as:**

1. Terrible
2. Very Poor
3. Poor
4. Neither good nor bad
5. Good
6. Very good
7. Excellent

[Theme: Course Satisfaction]

[Composite Score: Satisfaction]

**9. I learned new skills at the Surfers Rescue 24/7 course.**

- Strongly Disagree
- Disagree
- Somewhat Disagree
- Neither Agree nor Disagree
- Somewhat Agree
- Agree
- Strongly Agree
- Don't know / can't remember

[Theme: Course learning]

[Composite Score: Course learning]

**10. I remember most of what I learned at the Surfers Rescue 24/7 course.**

1. Strongly Disagree
2. Disagree
3. Somewhat Disagree
4. Neither Agree nor Disagree
5. Somewhat Agree
6. Agree
7. Strongly Agree

[Theme: Course Satisfaction]

[Composite Score: Satisfaction]

**11. I would recommend other surfers participate in a Surfers Rescue 24/7 course.**

1. Strongly Disagree
2. Disagree
3. Somewhat Disagree
4. Neither Agree nor Disagree
5. Somewhat Agree
6. Agree
7. Strongly Agree

[Theme: Course Satisfaction]

[Composite Score: Satisfaction]

**12. I have used what I learned from the Surfers Rescue 24/7 course in real life situations.**

- Strongly Disagree -> 13
- Disagree -> 13
- Somewhat Disagree -> 13
- Neither Agree nor Disagree -> 13
- Somewhat Agree -> 12a
- Agree -> 12a
- Strongly Agree -> 12a

[Theme: use and implementation of course skills/knowledge]

[Composite Score: Not Applicable]

**12A: How have you used the skills or knowledge you learned from Surfers Rescue 24/7?**

__________________________________ [Open text response]

[Theme: use and implementation of course skills/knowledge]

[Composite Score: Not Applicable]

**13. What was the most important lesson you learned from the Surfers Rescue 24/7 course (something you did not know before)?**

__________________________________ [Open text response]

[Theme: Course learning]

[Composite Score: Not Applicable]

**14. Completing the Surfers Rescue 24/7 course improved by ability to identify ocean/beach hazards and hazardous surf conditions.**

1. Strongly Disagree
2. Disagree
3. Somewhat Disagree
4. Neither Agree nor Disagree
5. Somewhat Agree
6. Agree
7. Strongly Agree

[Theme: Course learning]

[Composite Score: Course learning]

**15. Completing the Surfers Rescue 24/7 course improved by ability to recognize a swimmer or surfer in distress.**

- Strongly Disagree
- Disagree
- Somewhat Disagree
- Neither Agree nor Disagree
- Somewhat Agree -> 15a
- Agree ->15a
- Strongly Agree -> 15a

[Theme: Course learning]

[Composite Score: Course learning]

**15a. What are the signs that a person in the surf needs assistance?**

__________________________________ [Open text response]

[Theme: Knowledge check of course material]

[Composite Score: Not Applicable]

**16. Completing the Surfers Rescue 24/7 course improved my ability to safely rescue someone who needs help in the surf.**

1. Strongly Disagree
2. Disagree
3. Somewhat Disagree
4. Neither Agree nor Disagree
5. Somewhat Agree
6. Agree
7. Strongly Agree

[Theme: Course learning]

[Composite Score: Course learning]

**17. Today, I could rescue someone who needs help in the surf and keep myself safe while doing it.**

- Strongly Disagree
- Disagree
- Somewhat Disagree
- Neither Agree nor Disagree
- Somewhat Agree
- Agree
- Strongly Agree

[Theme: Confidence to perform rescue]

[Composite Score: Not Applicable]

**18. I believe that all surfers should complete a basic lifesaving and CPR course *(please select only one answer)***

1. Strongly Disagree
2. Disagree
3. Somewhat Disagree
4. Neither Agree nor Disagree
5. Somewhat Agree
6. Agree
7. Strongly Agree

[Theme: surfer role and responsibilities in coastal safety]

[Composite Score: surfer role and responsibilities in coastal safety]

**19. Did the Surfers Rescue 24/7 course change your opinion on the importance of surfers receiving basic rescue and/or CPR training?**

- Yes, my opinion changed
- No, my opinion is the same as before the course

[Theme: surfer role and responsibilities in coastal safety]

[Composite Score: Not Applicable]

**20. If a surfer (with a surfboard) rescues someone else, where should their surfboard be?**

- In between the beach and the victim
- In between the oncoming waves and the victim
- In between the surfer rescuer and the victim
- In between the surfer rescuer and the beach
- In between the surfer rescuer and the oncoming waves

[Theme: Knowledge check of course material]

[Composite Score: Not Applicable]

**21. As a surfer, I have a responsibility to look after the safety of others (both surfers and swimmers) in the water when I am surfing.**

1. Strongly Disagree
2. Disagree
3. Somewhat Disagree
4. Neither Agree nor Disagree
5. Somewhat Agree
6. Agree
7. Strongly Agree

[Theme: surfer role and responsibilities in coastal safety]

[Composite Score: Not Applicable]

**22. What is the most important component of a surfer's first response to a rescue situation? [Randomized answer order]**

- Ensuring your own safety and assessing conditions
- Reassuring the patient and explain the rescue plan
- Signalling for assistance
- Keeping the patient's face out of the water at all times
- Selecting the most appropriate rescue technique for the situation
- Don’t Know

[Theme: Knowledge check of course material]

[Composite Score: Not Applicable]

**22. Since you have completed the Surfers Rescue 24/7 program, have you rescued anyone from the ocean while you were surfing?**

***Rescue means that you physically helped them, for example giving them your board to float on, assisted them back to shore etc.***

- Yes -> 22a
- No -> 23

[Theme: use and implementation of course skills/knowledge]

[Composite Score: Not Applicable]

**22a. How many people have you rescued from the ocean since completing the course?**

***[Enter Number from a list]***

[Theme: use and implementation of course skills/knowledge]

[Composite Score: Not Applicable]

**22b. Please provide the following information on the person(s) your rescued since completing the Surfers Rescue 24/7 course?**

***[Display logic: Display the number of rows identified in 22a]***

|  | Do you believe the person would have drowned if you did not rescue them? *[Select one radio button]* | | Please indicate the approximate age of the person(s) you rescued. *[Dropdown for each row]* |
| --- | --- | --- | --- |
|  | Yes | No | Child (< 12)  Teen (12‐19)  Young Adult (approx. 20‐30)  Adult (30‐50)  Older Adult (51‐65)  Senior (65+) |
| Person 1 |  |  |  |
| Person 2 |  |  |  |
| Person 3 |  |  |  |
| Person 4 |  |  |  |

[Theme: use and implementation of course skills/knowledge]

[Composite Score: Not Applicable]

**23. In comparison to the time before you completed the Surfers Rescue 24/7 course, how often do you verbally warn swimmers or other surfers about ocean hazards or dangerous situations when you are surfing?**

*For example, tell swimmers about a rip current or submerged rocks.*

- Less often than before I completed the course
- About the same as before I completed the course
- More often since I completed the course

[Theme: use and implementation of course skills/knowledge]

[Composite Score: Not Applicable]

**24. I believe that the Surfers Rescue 24/7 course is an important initiative and it provides value to the community.**

1. Strongly Disagree
2. Disagree
3. Somewhat Disagree
4. Neither Agree nor Disagree
5. Somewhat Agree
6. Agree
7. Strongly Agree

[Theme: surfer role and responsibilities in coastal safety]

[Composite Score: Not Applicable]

**25. Since completing the Surfers Rescue 24/7 course, has your awareness of others who might be in trouble in the surf and on the beach changed?**

- Less aware than before I completed the course
- About the same as before I completed the course
- More aware since I completed the course

**26. Would you be interested in any of the following?**

***(Please check all that apply)***

- Another Surfers Rescue 24/7 course to refresh and practice skills
- An "Advanced" Surfers Rescue course that covers difficult/different rescue situations
- Training to a lifeguard/lifesaver level of surf rescue
- Becoming a patrolling lifeguard/lifesaver (Paid or volunteer)
- Being included in a community call-out team to respond to ocean emergencies in your local area

[Theme: Future opportunities]

[Composite Score: Not Applicable]

**27. Thank you for completing this survey! You are eligible to enter a random drawing to win a $500.00 voucher to Rip Curl. Would you like to enter this drawing?**

- Yes -> 27a
- No

[Theme: Survey Entry]

[Composite Score: Not Applicable]

**27a. To enter your name into a random drawing to win a $500.00 voucher to Rip Curl please provide the following contact details.**

First Name:

Last Name:

Phone Number:

Email:

Postal Address.

[Theme: Survey Entry]

[Composite Score: Not Applicable]

*[Survey Display logic: Show Q28 if Q22 = "Yes"]*

**28. Would you be willing to discuss your rescue(s) and experience with the Surfers Rescue 24/7 course with us? We will be hosting short, 20–30-minute online interviews to learn more about the influence of the course and the role that surfers play in emergency situations on the NSW coast. We will reimburse each interview participant with a $50 Coles gift card.**

***Note: Participation in these interviews is separate from the $500 Rip Curl Gift Card drawing and will not impact you inclusion or opportunity in that drawing.**

- Yes, the research team may contact me regarding an interview.
  - Name:
  - Email Address:
- No, I do not want to participate in an interview.

[Theme: Part Two Interview Recruitment]

[Composite Score: Not Applicable]
