# Supplementary material for: Impact of a surfer rescue training program in Australia and New Zealand: a mixed methods evaluation
Source: BMC Public Health. 2023 Nov 8;23:2193. doi: 10.1186/s12889-023-17057-w (PMC10631059; doi:10.1186/s12889-023-17057-w)
Supplement: Supplementary file 2 — Additional file 2. SR24/7 interview guide. [file 12889_2023_17057_MOESM2_ESM.docx]

**Additional File 2: SR24/7 Interview Guide**

Opening

- Introductions (Name, location, years surfing, surf locations, etc.)
- Objective of the interview
  - Our goal is to learn more about surfers' experiences taking the SR24/7 course and aiding people in the water who need help.
  - This information will help assess if the current program is working, and where/how it could be improved.
  - Your experience will help us understand what happens in emergency situations and how we can better support and improve safety on beaches in NSW
- Logistics
  - This interview is part of a research project conducted by the UNSW Beach Safety Research Group and Surfing Victoria/NSW/NZ.
  - No right or wrong answers
  - Interview should be about 30 minutes.
  - You were emailed a copy of the Participant Information and Consent Form
  - We are going to record the session, but will not use your name in any part of the project.
  - "Do you have any questions for me?"
  - "Do you consent to participate in this research and record the interview?"

Key Topic #1: SR24/7 Course

- What are the major reasons you decided to take the course?
- Please describe what you did during the Surfers Rescue 24/7 course.

Probing Questions:

- - Think back to the day you took the course, what sort of activities did you do? How did you feel? What did you learn that day?
- Overall, what did you think about the course?

Probing Questions:

- - Can you elaborate on what you liked about the course?
  - What recommendations do you have to improve the course?
  - How has the course influenced your surfing experience?
  - Would you and have you recommended the course to others?
  - What do you think are the challenges/barriers in encouraging other surfers to do the course? How should the course be promoted to surfers?
- Is there anything else about your experience with the course you would like to share?

Key Topic #2: Your Surfer Rescue

- Please think back to the day you made a rescue while you were surfing. Can you describe what happened by walking me through the entire event?

Probing questions:

- - Tell me about the conditions that day. (surf, weather, crowds, location)
  - How did you initially come to know that the person needed help? How did the situation develop?
  - How did you help the person(s)?
  - What happened to the person(s)?
  - In the moment when you were helping the person, how did you feel? What were you thinking / What was going through your head?
    - What about after the rescue was done? What were you feeling/thinking?
    - What about now, reflecting back on that rescue?
  - Did you use anything you learned in the course?

Is there anything else that you want to add about your rescue experience?

Closing

- Thank you for your time
- I will email you a copy of the transcript, if you want to correct or add anything please let me know.
